# Supplementary material for: Transmission of antimicrobial resistance in the gut microbiome of gregarious cockroaches: the importance of interaction between antibiotic exposed and non-exposed populations
Source: mSystems. 2023 Dec 14;9(1):e01018-23. doi: 10.1128/msystems.01018-23 (PMC10805027; doi:10.1128/msystems.01018-23)
Supplement: Supplemental Figures — Figures S1-S5. [file msystems.01018-23-s0001.pdf]

Supplementary Figures

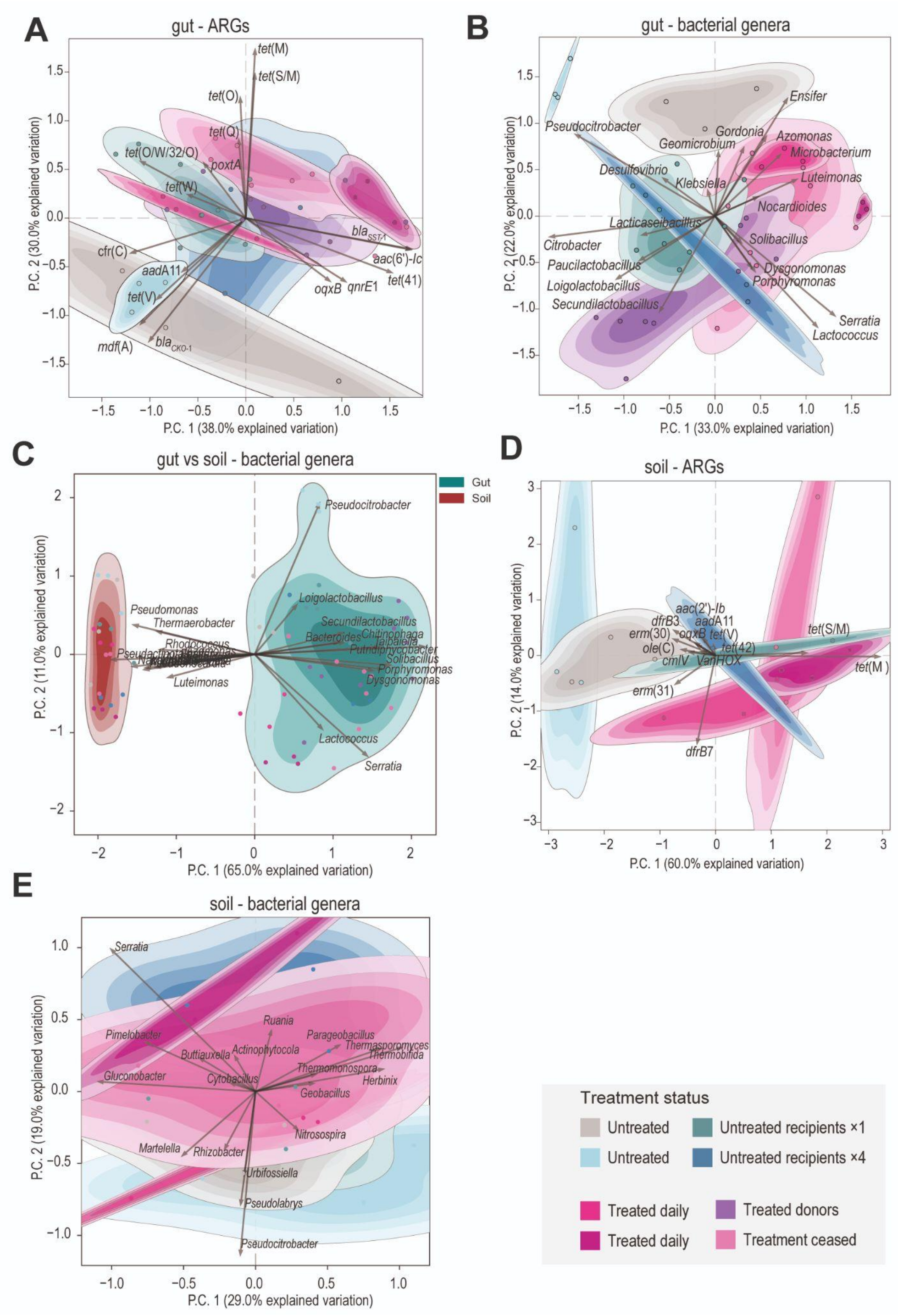

Figure S1. Ordination plots: Principal Component Analysis on CLR-transformed fragment counts. **A.** Differences in ARG composition between the gut microbiome samples (n=41). **B.** Differences in bacterial genera composition between the gut microbiome samples (n=41). **C.** Differences in bacterial genera composition between all microbiome samples (gut, n=41; soil, n=21). **D.** Differences in ARG composition between the soil microbiome samples (n=21). **E.** Differences in bacterial genera composition between the soil microbiome samples (n=21).

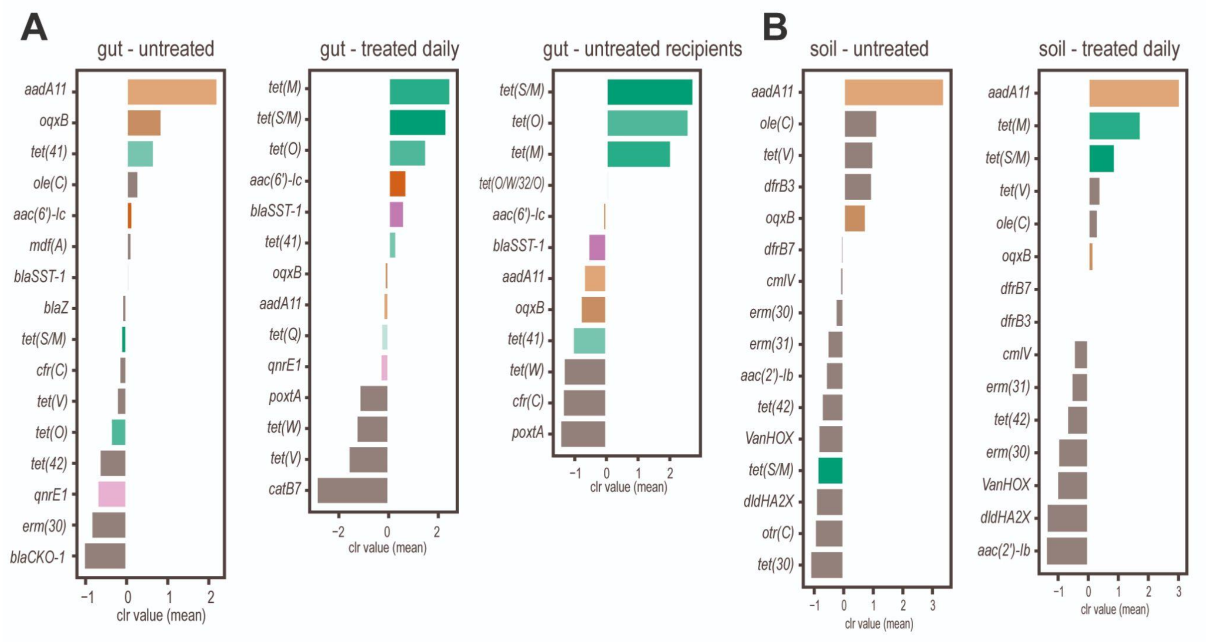

Figure S2. Relative abundance of the most abundant ARGS estimated as the mean CLR value for each ARG in each group. **A.** Gut microbiome samples: left - untreated microbiomes (day 8, n=3), middle - daily-treated microbiomes (day 8, 15, n=6), right - untreated recipients (day 15, n=6). **B.** Soil microbiome samples: left - untreated microbiomes (day 1, 8, n=6), right - daily-treated microbiomes (day 8, 15, n=6).

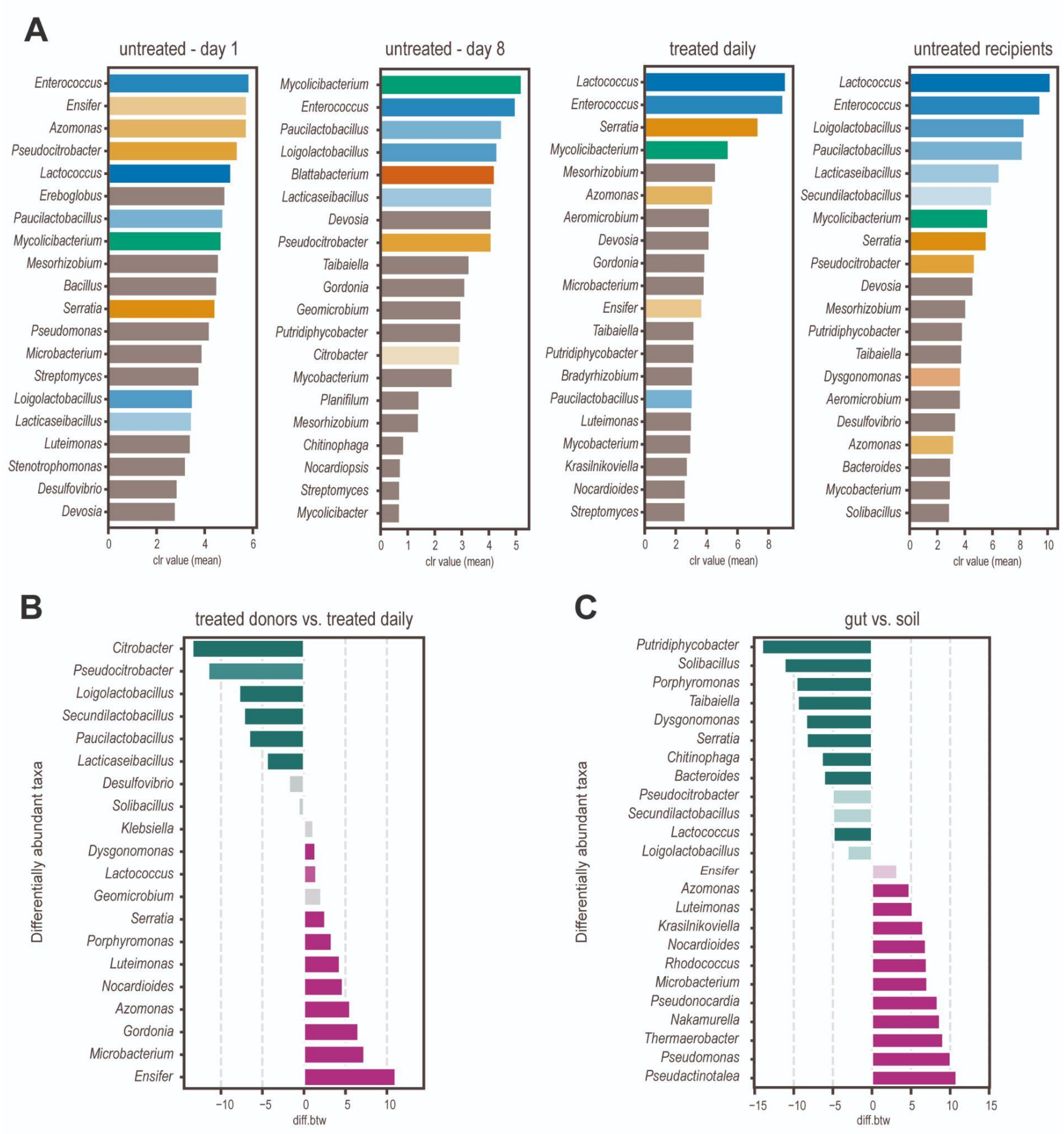

Figure S3. Relative abundance of the 20 most abundant bacterial genera in the gut microbiome, estimated as the mean CLR value for each genus in each group. **A.** From left to right: untreated microbiomes (day 1, n=3), untreated microbiomes (day 8, n=3), daily-treated microbiomes (day 8, 15, n=6), untreated recipients (day 15, n=6). B. Soil microbiome samples: left - untreated microbiome (day 1, 8, n=6), right - daily-treated microbiomes (day 8, 15, n=6). B, C. Differential abundance of bacterial genera between pairs of groups. Grey bars indicate |effect|<1, and lighter bars indicate wi.eBH > 0.05. **B.** Comparison between treated donors (day 10, 12, 15, n=9) and daily-treated (day 8 & 15, n=6) gut microbiomes. **C.** Comparison between gut (n=41) and soil (n = 21) microbiomes.

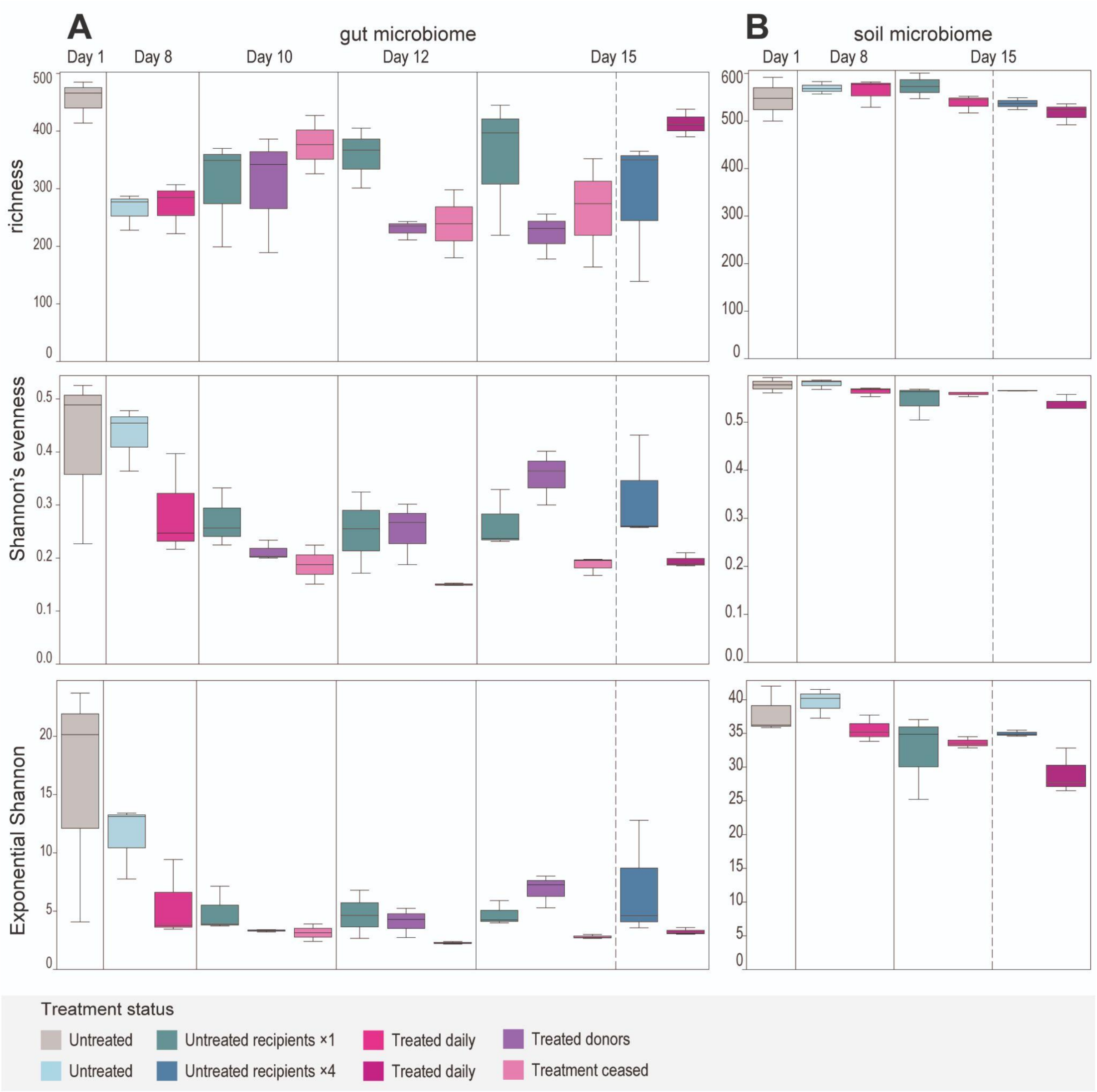

Figure S4. Bacterial diversity, as expressed in richness (top), Shannon's evenness (middle), and exponential Shannon index (i.e. effective number of genera) (bottom). **A.** Gut microbiome samples (n=41). **B.** Soil microbiome samples (n=21). Box colours indicate treatment status. All triplicates are ordered by day and treatment status.

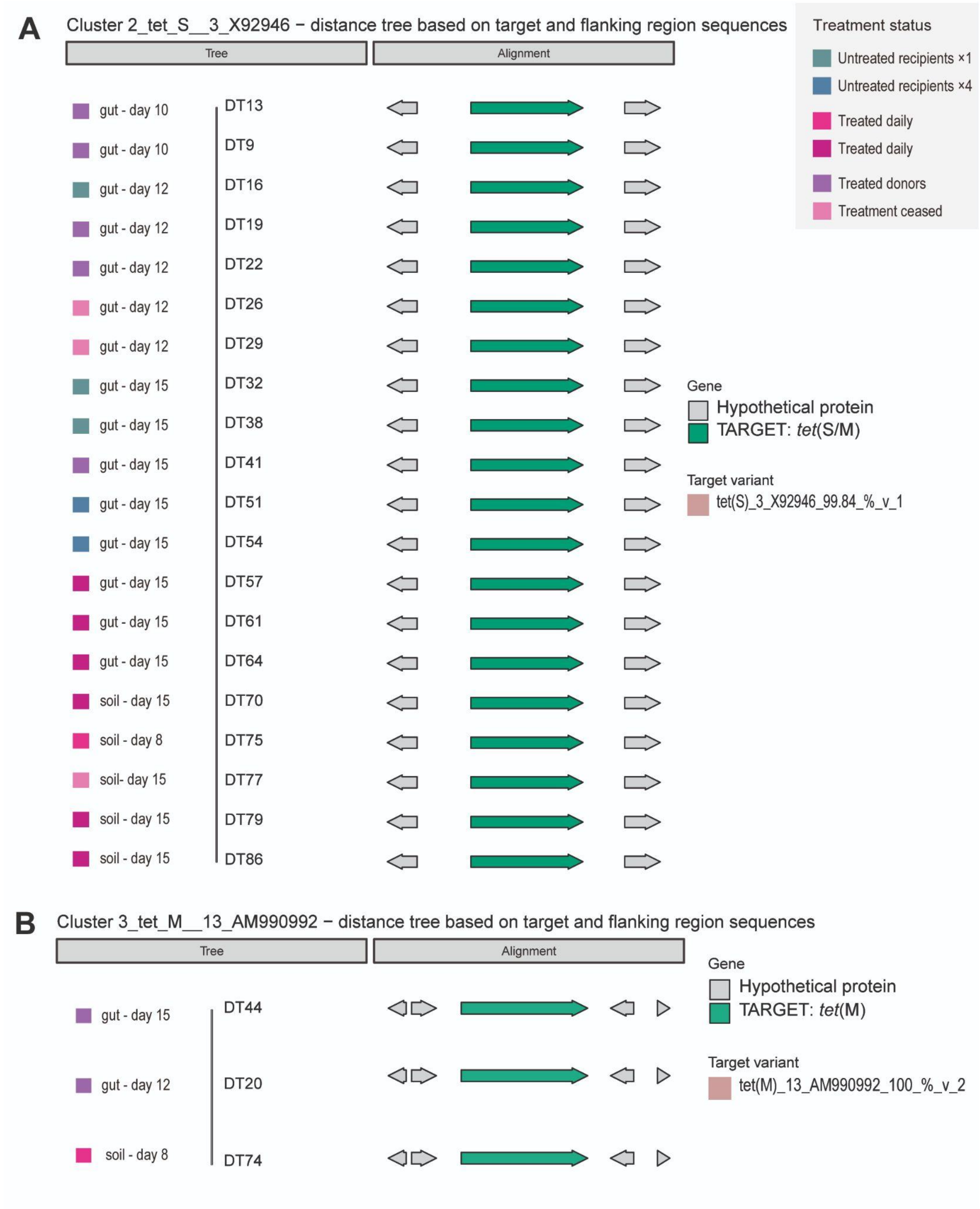

Figure S5. Flanking region analysis plots. Flank length was set to 1500 bp. A. Flanking regions of *tet*(S/M) gene. B. Flanking regions of *tet*(M) gene. Each gene and their flanking regions were identical across samples. The samples are colour coded based on treatment status.
